# A Comprehensive Phylogenetic and Structural Analysis of the Carcinoembryonic Antigen (CEA) Gene Family

**DOI:** 10.1093/gbe/evu103

**Published:** 2014-05-23

**Authors:** Athanasia Pavlopoulou, Andreas Scorilas

**Affiliations:** Department of Biochemistry and Molecular Biology, Faculty of Biology, University of Athens, Panepistimiopolis, Athens, Greece

**Keywords:** CEA, CEACAM, PSG, immunoglobulins, phylogeny, gene duplication

## Abstract

The *carcinoembryonic antigen* (*CEA*) gene family belongs to the immunoglobulin (Ig) superfamily and codes for a vast number of glycoproteins that differ greatly both in amino acid composition and function. The CEA family is divided into two groups, the carcinoembryonic antigen-related cell adhesion molecules (CEACAMs) and the pregnancy-specific glycoproteins. The CEA family members are implicated in pleiotropic (patho)physiological functions including cell–cell adhesion, pregnancy, immunity, neovascularization, regulation of insulin homeostasis, and carcinogenesis. In general, the *CEA*-encoded proteins are composed of an extracellular region with Ig variable and constant-like domains and a cytoplasmic region containing signaling motifs. Of particular interest, the well-studied human and mouse *CEA* genes are arranged in clusters in a single chromosome. Taking into account this characteristic, we made an effort to reconstruct the evolutionary history of the *CEA* gene family. Toward this end, the publicly available genomes were searched extensively for *CEA* homologs. The domain organization of the retrieved protein sequences was analyzed, and, subsequently, comprehensive phylogenetic analyses of the entire length CEA homologous proteins were performed. A series of evolutionarily conserved amino acid residues, functionally important, were identified. The relative positioning of these residues on the modeled tertiary structure of novel CEA protein domains revealed that they are, also, spatially conserved. Furthermore, the chromosomal arrangement of *CEA* genes was examined, and it was found that the *CEA* genes are preserved in terms of position, transcriptional orientation, and number in all species under investigation.

## Introduction

The *carcinoembryonic antigen* (*CEA*) gene family, which belongs to the immunoglobulin (Ig) gene superfamily, comprised an exceptionally diverse array of highly glycosylated glycoproteins (Paxton et al. 1987; Zhou et al. 2001). The CEA family is broadly divided into two groups, the CEA-related cell adhesion molecules (CEACAMs) and the pregnancy-specific glycoproteins (PSGs) (Hammarstrom 1999). In humans, based on our current phylogenetic analysis, the CEA family consists of 35 genes, 21 of which are protein coding, arranged in contiguous clusters in chromosome 19 in the region 19q13.2-19q13.4 (Hammarstrom 1999).

The *CEA*-encoded proteins have varying length and domain organization, which probably reflects their functional divergence. All currently reported CEA-encoded proteins consist of at least one Ig variable (IgV)-like domain, followed by a varying number of Ig constant (IgC)-like domains (Brummendorf and Rathjen 1995). The core structure of these domains, the Ig-like fold, is characterized by two β-sheets (faces) that cross over each other. The IgV-like domain, approximately 110 amino acids long, contains a conserved basic (arginine) and an acidic (aspartate) amino acid, which are proposed to stabilize the Ig-like fold via an intradomain salt bridge. The CFG-face of the IgV-like domain (named after the C-F-G strands it is composed of) mediates homotypic and heterotypic cell–cell adhesion (Taheri et al. 2000). The IgC-like domain, contains two conserved cysteine residues, that occupy the corresponding positions of arginine and aspartate, stabilize the Ig-like conformation by forming a disulphide bridge (Williams and Barclay 1988; Bork et al. 1994).

*CEACAM* genes are expressed in a wide variety of cell types including epithelial, endothelial, and immune cells such as leukocytes and dendritic cells, whereas *PSGs* are expressed exclusively in the placental trophoblasts (Hammarstrom 1999). CEACAMs are either inserted into the cell membrane via a transmembrane (TM) domain or they are linked to the membrane via semipenetrating glycosylphosphatidylinositol (GPI) anchorage (Naghibalhossaini et al. 2007). The latter type of membrane anchorage has been detected only in primates thus far. The membrane-bound CEACAMs possess a C-terminal cytoplasmic domain, which may contain motifs associated with signal transduction (Hammarstrom 1999).

Members of the CEA family are implicated in diverse physiological and pathological functions (Obrink 1997; Kuespert et al. 2006). For instance, CEACAMs play a vital role during embryonic development where cell–cell adhesion is necessary to integrate the cells into functional organs (Kuespert et al. 2006). Members of the CEACAM group also serve as receptors of several bacterial and viral pathogens, such as the murine hepatitis virus, *Haemophilus influenza*, *Neisseria meningitides,* and *N. gonorrhea,* which bind CEACAM proteins via their N-terminal IgV-like domain (Bos et al. 1999; Virji et al. 1999, 2000; Villullas et al. 2007). PSGs are secreted proteins from fetal trophoblasts, which are proposed to regulate the maternal–fetal interactions during pregnancy (Hau et al. 1985; Ha et al. 2010). Of particular note, CEA play an important role in carcinogenesis (Scorilas et al. 2003; Michaelidou et al. 2013). The prototypic member of this family, human CEA (henceforth referred to as CEACAM5), was discovered by Gold and Freedman (1965) in the mid-1960s in the blood of patients with colon cancer. CEACAM5 is consistently overexpressed in various malignancies frequently associated with poor patients’ clinical outcome and reduced overall survival (Chevinsky 1991). These properties have made CEACAM5 a prominent clinical cancer biomarker, widely used in early diagnosis, effective prognosis, and monitoring of colon cancer, as well as other types of cancers (Gaglia et al. 1988; Ballesta et al. 1995).

In this study, we made an effort to reconstruct the evolutionary history of *CEA* gene family and identify conserved amino acids that may play important role in the overall structure and function of CEA proteins. To this direction, the fully sequenced and the nearly complete sequenced genomes were searched for *CEA* homologs. Members of the *CEA* family were identified in diverse taxa covering an evolutionary range from cartilaginous fishes to human. Subsequently, comprehensive phylogenetic analyses were performed employing the maximum likelihood (ML) and the neighbor-net methods. The genomic arrangement of the identified *CEA-*related genes was also analyzed, and it was shown that in different species, these genes are arranged in contiguous clusters with conserved position and orientation of transcription. On the basis of both the phylogenetic and syntenic analyses, we identified eight conserved gene clusters. Furthermore, the protein domain organization of the CEA homologs was examined and amino acid conservation patterns were identified. The three-dimensional (3D) structure of domains from species of the basal taxonomy was predicted with homology modeling, and the evolutionarily conserved amino acids were mapped onto these structures.

## Materials and Methods

### Sequence Database Searching

The names or accession numbers of the characterized CEA reported in literature were used initially to retrieve their corresponding sequences from the publicly available nonredundant sequence databases ENSEMBL (Flicek et al. 2013), National Center for Biotechnology Information (NCBI)’s RefSeq (Pruitt et al. 2012), and UniProtKB (Magrane and UniProt Consortium 2011). To obtain more putative CEA homologs, these sequences were used subsequently as probes to perform extensive reciprocal BLASTp and tBLASTn (Altschul et al. 1990) searches of genomes with high coverage (>6×) and low coverage (2×). This process was reiterated until convergence, that is, no novel putative CEA sequences could be detected. The longer known transcript was selected. The partial or ambiguous sequences were not included in the subsequent steps of the study. The Translate program (http://web.expasy.org/translate/, last accessed May 27, 2014) was used to translate nucleotide sequences.

### Motifs Construction

Representative CEA peptide sequences were aligned with MAFFT v.7 (Katoh and Standley 2013, 2014) and edited with Utopia suite’s CINEMA alignment editor (Pettifer et al. 2009). Sequence motifs were excised from the multiple sequence alignments, manually edited for insertions or gaps. They were submitted to WebLogo3 (Crooks et al. 2004) with default options, to generate consensus sequences.

### Chromosomal Localization

The chromosomal localization of the *CEA* genes was determined using the ENSEMBL GeneView (Flicek et al. 2013) and the NCBI MapViewer (Wolfsberg 2011).

### Alignments and Phylogenetic Analyses

The full-length CEA amino acid sequences were aligned with MAFFT v.7. The resulting multiple sequence alignments were edited using CINEMA alignment editor (Pettifer et al. 2009). The trimmed alignments were then used to reconstruct phylogenetic trees by employing two separate methods. To obtain ML-based trees, the method implemented in the software package MEGA, version 5.2 (Tamura et al. 2011) was used. In this study, a distance-based tree (BIONJ) (Gascuel 1997) was used as seed, as well as the nearest-neighbor-interchange heuristic with five discrete gamma categories of evolutionary rates. The number of amino acid substitutions per position was estimated with the JTT model (Jones et al. 1992). Trees were also reconstructed employing the neighbor-net method (Bryant and Moulton 2004) implemented in SplitsTree v.4 (Huson 1998; Kloepper and Huson 2008), a distance-based method able to detect conflict between phylogenetic signals in the form of networks; the Ucorrected P model of substitution was used. For both methods, bootstrap analyses (200 pseudoreplicates) were conducted to evaluate the statistical significance of the reconstructed trees. The trees generated with the ML method were illustrated with Dendroscope v.3 (Huson and Scornavacca 2012).

### Protein Domain Organization

The consensus boundaries of the individual protein domains in CEA proteins were determined from the full-length CEA amino acid sequences combining the outputs of the search engines available in SMART v.7 (Letunic et al. 2012), PFAM v.27 (Punta et al. 2012) and CDD v.3 (Derbyshire et al. 2012) and InterPro v.42 (Hunter et al. 2012) protein signature databases. The TM regions were predicted with the programs MINNOU (Cao et al. 2006) and PRED-TMR2 (Pasquier et al. 1999).

### Homology Modeling

The 3D structures of the IgV-like domain of MedakaCea, FrogCea7, and LizardCeacam19, and the 3D structure of the IgC-like domain of the FrogCea2 and PlatypusCea1 (target proteins) were predicted by homology modeling. The X-ray crystal structures of the murine Ceacam1a (PDB ID: 1L6Z) (Tan et al. 2002) and the human CEACAM1 (PDB: 2GK2) (Fedarovich et al. 2006) were used as templates to model the IgC-like and IgV-like domain, respectively, with the modeling package Modeller (Sali et al. 1995). To remove any local constraints, the generated protein models were subjected to energy minimization using the Charmm27 forcefield, implemented in Gromacs v.4.5.5 (Hess et al. 2008). The quality of the final modeled protein structures was evaluated using Procheck (Laskowski et al. 1996) and ANOLEA (Melo et al. 1997). The protein models were illustrated with PyMol (DeLano 2002). Furthermore, the secondary structure of the TM domain of the sequences ZebrafishCea1, FrogCea7, LizardCeacam19, and HumanCEACAM1 was predicted using the bioinformatics tools described in Pavlopoulou and Michalopoulos (2011). The predicted TM helices were modeled, template free, as described above.

## Results

### Identification of *CEA* Homologs

In this study, we performed comprehensive and updated phylogenetic analyses of the *CEA* homologs in the available genomes of 33 species: *Anolis carolinensis* (lizard), *Bos taurus* (cow), *Branchiostoma lancelatum* (amphioxus), *Callithrix jacchus* (marmoset), *Canis familiaris* (dog), *Ciona intestinalis* (ascidia), *Danio rerio* (zebrafish), *Dasypus novemcinctus* (armadillo), *Drosophila melanogaster* (fruit fly), *Equus caballus* (horse), *Gallus gallus* (chicken), *Homo sapiens* (human), *Latimeria chalumnae* (coelacanth), *Lepisosteus oculatus* (spotted gar), *Leucoraja erinacea* (little skate), *Loxodonta africana* (elephant), *Macaca mulatta* (macaque), *Microcebus murinus* (mouse lemur), *Monodelphis domestica* (opossum), *Mus musculus* (mouse), *Myotis lucifugus* (microbat), *Ornithorhynchus anatinus* (platypus), *Oryzias latipes* (medaka), *Otolemur garnettii* (bushbaby), *Pan troglodytes* (chimpanzee), *Pelodiscus sinensis* (Chinese softshell turtle), *Petromyzon marinus* (lamprey), *Pongo abelii* (orangutan), *Rattus norvegicus* (rat); *Taeniopygia guttata* (zebra finch); *Takifugu rubripes* (pufferfish), *Tupaia belangeri* (tree shrew), and *Xenopus tropicalis* (frog). The genomes with high coverage were selected to avoid underestimation of the number of *CEA* genes, like in the case of low coverage genomes. Collectively, 207 *CEA* protein-encoding genes, 13 pseudogenes, and 1 expressed sequence tag (EST) sequence were identified in the genomes of 20 species representing diverse eukaryotic taxonomic divisions (according to the NCBI taxonomy database [Federhen 2012]) (supplementary table S1, Supplementary Material online) rimates (87), rodentia (47), perissodactyla (9), cetartiodactyla (6), carnivore (10), afrotheria (5), xenarthra (7), metatheria (15), proteotheria (5), sauria (2), amphibia (13) and teleosts (14), and chondrichthyes (1). Despite extensive database searches, *CEA* homologs were not detected in the complete and well-annotated genomes of aves, insects, and in lower vertebrates such as craniata, cephalochordata, and ascidia.

To prevent confusion, we used the revised nomenclature by Beauchemin et al. (1999), for human and rodent CEA sequences; for the rest, we used the names provided in the original references and the sequence databases. Regarding the newly identified sequences (e.g., primate CEA), they were named by virtue of homology to their closest related well-annotated human and mouse CEA genes. The distant homologs (e.g., frog and fish CEA), with no significant sequence similarity to the known CEA, were commenced by CEA followed by an ascending number depending on their order in the chromosome.

### Conserved Structural Features of CEA Proteins

**Fig. 1.—:**
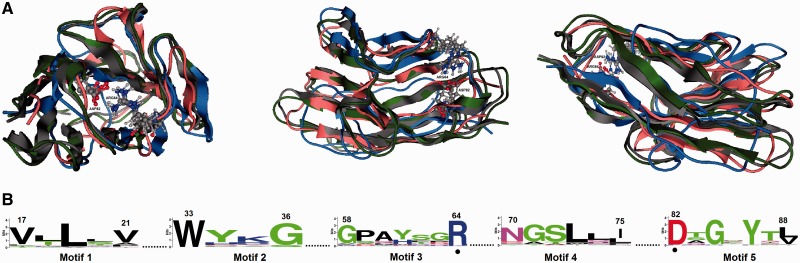
IgV-like domain. (*A*) Homology models of the IgV-like domain of MedakaCea (pink), FrogCea7 (green), and LizardCeacam19 (blue) in cartoon representation superposed on the human CEACAM1 (PDB ID: 2GK2) (gray). The residues arginine and aspartate that form a salt bridge are shown as a ball-and-stick representation. (*B*) The conserved proteins motifs derived from the IgV-like domain. The amino acid residue numbers (according to human CEACAM1) are indicated. The invariant residues arginine and aspartate are indicated by dots. The letters, representing amino acid residues of the motif sequences, are piled one on top of another at every position in the sequences. The height of each letter is proportional to the frequency of the corresponding amino acid at that position; the letters are ordered, so the most frequent one is on the top. The height of the whole pile is normalized, so that it indicates the information content (measured in bits) in each position.

**Fig. 2.—:**
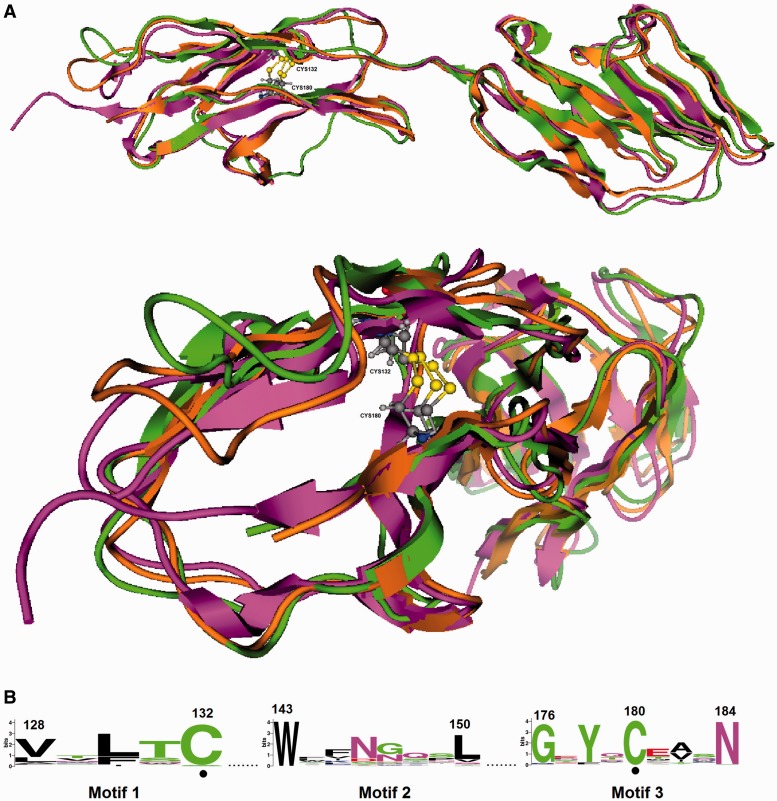
IgC-like domain. (*A*) Modeled protein structures of the IgC-like domain of FrogCea2 (green) and PlatypusCea1 (brown) superimposed onto the murine Ceacam1a (PDB ID: 1L6Z) (purple). The cysteines involved in the formation of the disulfide bridge are shown as a ball-and-stick representation, and the disulfide bridges are indicated by yellow lines. (*B*) The conserved proteins motifs are detected in the IgC-like domain, numbered according to murine Ceacam1a. The invariant cysteine residues are denoted by dots.

**Fig. 3.—:**
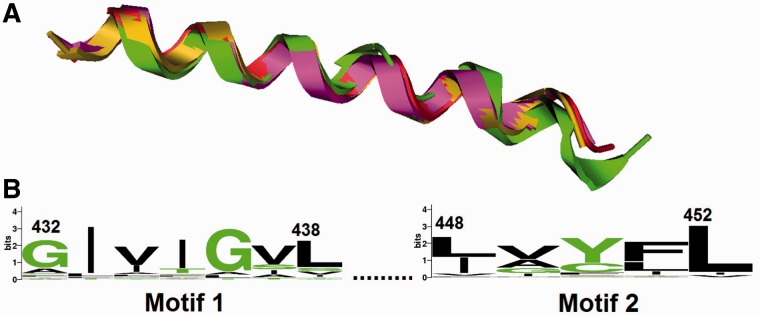
TM domain. (*A*) Modeled TM helices of HumanCEACAM1 (purple), LizardCeacam19 (gold), FrogCea7 (green), and ZebrafishCea1 (red). (*B*) The conserved TM protein; the amino acid numbering is based on HumanCEACAM1.

**Fig. 4.—:**
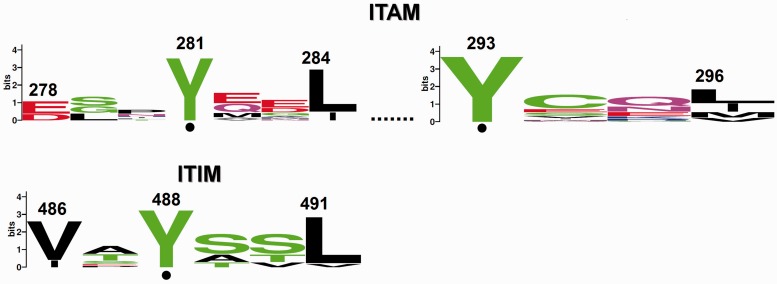
Cytoplasmic domain. ITAM and ITIM. The invariant tyrosine residues are indicated by dots, numbered according to HumanCEACAM19 and MouseCeacam1, respectively.

The CEA homologous proteins were found to differ greatly in their length and domain organization. On the basis of the combined output of the signature databases and the multiple alignment of CEA protein sequences, we determined the organization of the three major protein domains in the extracellular region of the CEA proteins, namely IgV-like, IgC-like, and TM, the immunoreceptor tyrosine-based activation motif (ITAM) and immunoreceptor tyrosine-based inhibition motif (ITIM) in the cytoplasmic region and the GPI anchors. Furthermore, consensus protein motifs were derived from the multiple alignment of sequences that correspond to the three extracellular domains and the cytoplasmic domain and a number of conserved amino acid residues were identified (figs. 1–4).

Given that the 3D structure of a protein is more conserved than its corresponding amino acid sequence, an effort was made to map the position of these residues to the tertiary structure of representative CEA domains. Toward this end, the 3D structure of the IgV-like (fig. 1) and IgC-like (fig. 2) domains from putative, evolutionarily diverse CEA proteins were predicted with homology modeling using the resolved crystal structures of the IgV-like domain of the human CEACAM1 (PDB ID: 2GK2) and the murine Ceacam1a (PDB ID: 1L6Z) as templates, respectively.

As shown in the superimposed structures of the MedakaCea, FrogCea7, and LizardCeacam19 IgV-like domains (fig. 1), the major secondary structures are conserved. The residues arginine (R64) and aspartate (D82) that form a salt bridge are also conserved in the modeled protein structures. Also, the amino acid asparagine (N70), suggested to be involved in glycosylation, was found to be highly conserved.

As shown in figure 2, the modeled 3D structures of the IgC-like domain of FrogCea2 and PlatypusCea1 superimposed onto the N-terminus of the murine Ceacam1a exhibit notable similarity in their secondary structure elements. The two invariant cysteine residues, which are involved in the formation of the disulfide bridge, were found to be spatially conserved in the IgC-like domain of the CEA homologs (fig. 2).

The TM domains of the homologous CEA proteins were predicted to adopt an α-helical conformation (fig. 3). Two prime signature motifs were also identified in the TM domain.

In the cytoplasmic region, consensus ITAM and ITIM were identified, where the tyrosine residue is invariant (fig. 4). Phosphorylation of ITAM/ITIM initiates of terminates, respectively, signal transduction pathways implicated in cellular proliferation (Beauchemin et al. 1997) or regulation of immune response.

### Syntenic Mapping of *CEA* Homologous Genes

**Fig. 5.—:**
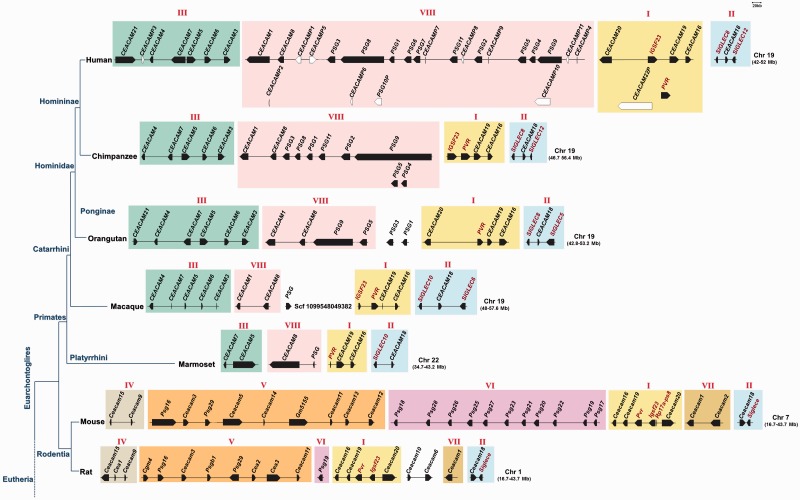

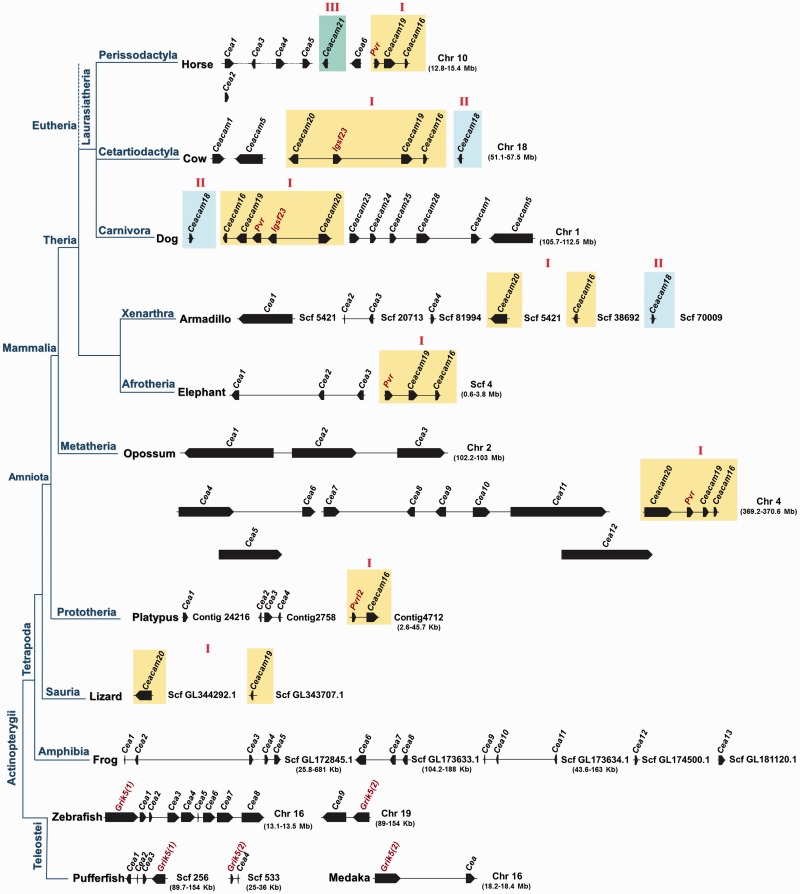
Schematic depiction of the chromosomal arrangement of *CEA* genes. The orientation of transcription and approximate position and size of *CEA* genes are indicated. The genomic boundaries of each chromosome/scaffold/contig are shown in parentheses. The *CEA* protein encoding genes are shown as filled arrowheads, and the *CEA* pseudogenes are indicated by open arrowheads. The non-*CEA* genes flanking the *CEA* genes are shown in dark red. The *CEA* gene clusters are indicated by roman numerals and different coloration. An NCBI-derived cladogram illustrating the evolutionary relationships of the taxa under study is shown on the left. Chr: chromosome; Scf: scaffold.

The chromosomal arrangement of the *CEA* homologous genes found in the genomes of all species under study was investigated. As shown in figure 5, the homologous *CEA* genes are arranged in clusters, with conserved sequential order, transcriptional orientation, number, and flanking genes, in all species under investigation—at least in the high-coverage genomes. We identified eight (I–VIII) conserved gene clusters in our study, which are indicated by roman numerals and numbers according to the order of their appearance in the evolutionary timetable (fig. 5). The “ancestral” Cluster I appeared first in the common ancestor of extant amniotes and contains the genes *CEACAM20*, *CEACAM19*, and *CEACAM16*. Cluster II was emerged in the common ancestor of extant eutherians for the first time and contains a single gene, *CEACAM18*. Cluster III appeared in the common ancestor of euarchontoglires and laurasiatheria for the first time (*CEACAM21*). Subsequent duplications of *CEACAM21* have apparently given rise to *CEACAM3–7* in the primate lineage. Clusters IV–VII are restricted to the glire lineage and more specifically to rodents. Cluster VIII is primate specific because it was detected only in primates. Of particular note, the *CEA* homologs of the New World monkeys are localized on chromosome 19. Because of incomplete genomic studies, the *CEA* genes of several organisms were detected in chromosomal fragments. Therefore, in this study, a *CEA* member was considered to be absent both if the gene was not detected and the *CEA* genes that flank it in the prototypic human and mouse sequential order are detected in the same chromosome, scaffold, or contig (fig. 5).

### Phylogenetic Analyses

**Fig. 6.—:**
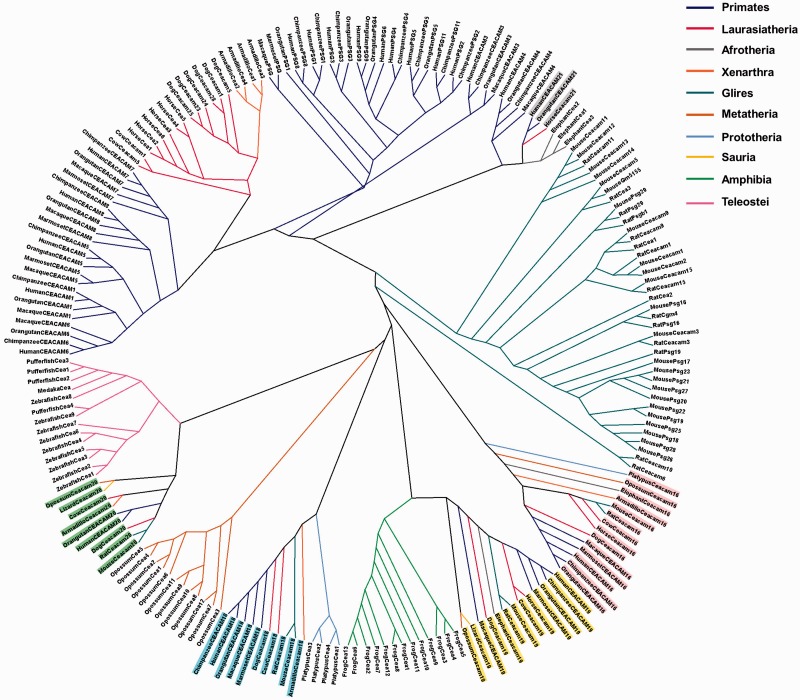
ML radial cladogram of CEA proteins. The sequences are represented by the species name and the CEA protein name. The branches are colored according to the eukaryotic taxa. CEACAM16, CEACAM18, CEACAM19, CEACAM20, and CEACAM21are highlighted by different shading.

**Fig. 7.—:**
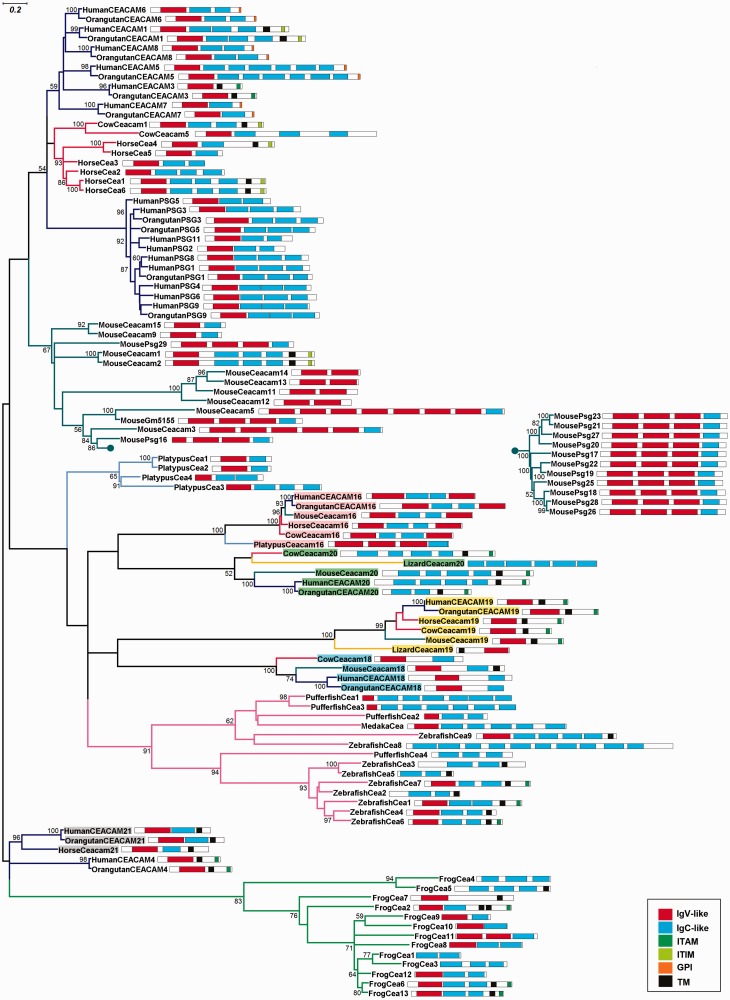
ML phylogram of representative CEA proteins. For clarity, the mouse Psg clade (Cluster VI) is condensed and shown separately. Bootstrap values greater than 50% are indicated at the nodes. The branch lengths depict evolutionary distance. The domain organization of CEA proteins is presented on the right of the corresponding sequences. The domain legends are shown in the figure inset. The scale bar at the upper left indicates the length of amino acid substitutions per position. The conventions are the same as in figure 6.

To investigate the evolutionary relationships among CEA, comprehensive phylogenetic analyses based on the entire length protein sequences of all species under study were conducted. Two different methods for phylogenetic reconstruction, ML and neighbor-net, were employed to resolve better the evolutionary relationships. The trees generated with both methods are congruent as their overall topology is similar (fig. 6 and supplementary fig. S1, Supplementary Material online). Representative CEA sequences of selected species with complete or almost complete genomes were selected for more accurate phylogenetic analysis, using both tree construction methods (fig. 7 and supplementary fig. S2, Supplementary Material online). The low support values (below 50) in some nodes suggest alternative branching patterns.

The CEACAM16, CEACAM18, CEACAM19, and CEACAM20 homologs form their own distinct clades with representatives from almost every taxonomic division (figs. 6 and 7, supplementary figs. S1 and S2, Supplementary Material online). *CEACAM19* and *CEACAM20* appear to be the primordial genes of the *CEA* family (fig. 5) because they were found in the common ancestor of amniotes. However, neither C*EACAM19* nor *CEACAM20* orthologs were identified in prototheria (platypus) (fig. 5); this is probably due to incomplete genomic studies. *CEACAM16* was detected for first time in prototheria, whereas *CEACAM18* appeared later in evolution in the common ancestor of extant eutherians. CEACAM21 orthologs also forms a coherent clade (figs. 6 and 7, supplementary figs. S1 and S2, Supplementary Material online). Interestingly, CEACAM21, which was detected first in the order of perissodactyla and then again in the superfamily of apes (fig. 5), is restricted to three species, namely human, orangutan, and horse. This finding triggers the speculation that either a *CEACAM21* gene may have existed in other species, which was deleted during the course of evolution, or *CEACAM21* evolved independently within these three species.

*CEACAM1, 3–8* were found only in primates (fig. 5). In particular, CEACAM5, CEACAM7, and CEACAM8 appeared in the common ancestor of New World monkeys (marmoset) for first time whereas CEACAM1, CEACAM3, CEACAM4, and CEACAM6 arose later in Old World monkeys (macaque) (fig. 5). CEACAM1 and CEACAM3–8 appear to form separate monophyletic branches (albeit moderately supported) (figs. 6 and 7, supplementary figs. S1 and S2, Supplementary Material online), leading to the suggestion that primate-specific *CEACAM1, 3–8* gene duplications must have taken place. The domain organization of CEACAM1, 3–8 is also preserved among species (fig. 7). The GPI anchor was detected only in primates CEACAM5–8 corroborating, in this way, previous reports (Naghibalhossaini and Stanners 2004).

Based on the phylogenies (figs. 6 and 7, supplementary figs. S1 and S2, Supplementary Material online), the *PSG* genes of primates and the corresponding *Psg* genes of rodents form two different monophyletic branches, leading to the suggestion that *PSG* and *Psg* genes have likely expanded after the divergence of primates and rodents. Given that the PSG protein sequences of apes cluster with the corresponding PSGs of the fellow apes and not with the PSGs of their own species (e.g., human, chimpanzee, and orangutan PSG3) (figs. 6 and 7, supplementary figs. S1 and S2, Supplementary Material online) along with the observation that both their length and domain organization are different (fig. 7), we speculate that the PSGs of apes were derived from duplication events that have presumably preceded the speciation of apes. On the other hand, the PSGs of the New World monkeys form a subclade within the PSG clade (fig. 6 and supplementary fig. S1, Supplementary Material online), suggesting that they have rather evolved independently of those in apes.

As opposed to primate *PSGs*, a series of species-specific gene duplications must have occurred in rodents yielding 11 *Psg* paralogs (Cluster VI) (fig. 5) in mouse, which share significant sequence similarity (figs. 6 and 7 and supplementary figs. S1 and S2, Supplementary Material online). Regarding the domain organization, mouse Psg proteins harbor three IgV-like domains, whereas primate PSGs possess only one (fig. 7). In the rodent-specific Cluster VII (fig. 5), *Ceacam1* and *Ceacam2* are likely the products of a tandem gene duplication subsequent to the mouse–rat divergence because mouse Ceacam1 and Ceacam2 cluster together with high confidence (figs. 6 and 7, supplementary figs. S1 and S2, Supplementary Material online). However, this is not the case in the genes located in Clusters IV and V (fig. 5), which appear to have expanded prior to the rodent speciation (figs. 6 and 7, supplementary figs. S1 and S2, Supplementary Material online).

The *Cea* homologs that were identified in zebrafish and pufferfish are located in two different chromosomal fragments flanked by the *Grik5* co-orthologs, *Grik5*(*1*) and *Grik5*(*2*) (fig. 5). Given that a whole-genome duplication occurred in teleost fishes subsequent to their divergence from nonteleost ray-finned fishes, approximately 320–400 Ma (Hoegg et al. 2004; Jaillon et al. 2004; Meyer and Van de Peer 2005; Kasahara et al. 2007), it would be reasonable to suggest that the zebrafish and pufferfish *Cea* are probably the products of this teleost-specific duplication.

According to the phylogenetic trees (figs. 6 and 7, supplementary figs. S1 and S2, Supplementary Material online), the teleost Cea protein sequences cluster in a well-supported monophyletic clade (with a bootstrap value of 91) (fig. 7). Therefore, the *Cea* genes detected in the contemporary teleost genomes must have been derived from a series of lineage-specific duplications, as in the case of amphibian, prototherian, metatherian, and specific therian *CEA*-related genes. This hypothesis is also supported by the relatively large evolutionary distances and the diverse domain organization of the proteins encoded by the above genes (fig. 7).

## Discussion

Several experimental studies have focused on the expansion of *CEA* in specific species or taxa (Zhou et al. 2001; McLellan et al. 2005; Zebhauser et al. 2005; Weichselbaumer et al. 2011). In a more recent experimental effort, several *CEA-*related genes were also detected in vertebrates (Chang et al. 2013). The availability of a growing number of sequenced genomes enabled us to perform, for the first time, comprehensive phylogenetic and structural analyses of CEA. In this study, *CEA* members were identified in organisms from different taxonomic divisions, ranging from cartilaginous fishes to humans. An EST sequence was detected in little skate (*L. erinacea*), which was found to be a CEA homolog based on BLAST searches. This allowed us to trace the evolutionary origin of *CEA* approximately 450–420 Ma when chondrichthyes emerged (Venkatesh et al. 2014). A large number of *CEA* members were detected in teleosts, frog, platypus, opossum, elephant, armadillo, dog, and horse without any homologs from other species, suggesting lineage/species-specific gene amplification. PSG homologs were detected exclusively in the superorder of euarchontoglires (primates and rodents), which have hemochorial placentae (Carter and Enders 2004) and not in other mammalian orders with different type of placentation such as epitheliochorial or endotheliochorial (Zeiler et al. 2007). On the basis of this finding, we could suggest that *PSGs* have expanded after the radiation of euarchontoglires to perform functions related to the hemochorial mode of placentation.

Subsequently, phylogenetic reconstructions were performed with the entire length of the *CEA*-encoded proteins to include all the available evolutionary information that is present in the amino acid sequences. In this way, a series of sequentially and spatially conserved amino acids were also identified in the IgV- and IgC-like domains. The conservation of these residues across the diverse CEA family members suggests the importance of these residues in the overall structure and function of CEA.

In this study, the chromosomal arrangement of the *CEA* homologs in all species under investigation was examined. A prominent feature of the *CEA* gene family is that it consists of clusters of genes with conserved order and orientation, mapped to a single chromosome, in all species. On the basis of both syntenic and phylogenetic analyses, we identified a total of eight (I–VIII) conserved gene clusters, the first one appearing for first time in the common ancestor of amniotes. Moreover, the flanking non-*CEA* genes such as *Grik5*, *PVR*, *SIGLECs*, and *IGSF23* are also conserved with the same order and position in all organisms under study. Given that shared synteny is likely associated with function (Wang et al. 2008), we suggest that these genes may have evolved along with *CEA* to complement *CEA’s* function.

The *CEA* gene family represents a notable example of gene duplication, a process suggested to be essential for the development of novel genes (Demuth et al. 2006). The extensive presence of duplicated genes such as *kallikreins* (Pavlopoulou et al. 2010), *bitter taste receptor* (*T2R*) genes, mammalian *lysozyme* gene family (Dong et al. 2009), genes encoding for keratin associated proteins (KRTAPs) (Wu et al. 2008), and the *oxidative phosphorylation* (*OXPHOS*) gene families (De Grassi et al. 2008), all of which are implicated in important physiological processes, points out the importance of this process. We assume that successive rounds of gene duplications, followed by deletions, inversions, translocations, and divergence have likely given rise to the *CEA* genes found in the contemporary genomes.

Of particular note, both ITAM and ITIM were detected together in human, orangutan, mouse, horse, and cow CEACAM proteins. This observation leads to the suggestion that evolutionary pressure could have applied to ITAM and ITIM, motifs exerting opposing signaling effects (activating vs. inhibitory), to coevolve (Kammerer and Zimmermann 2010). In particular, recognition of bacterial pathogens by CEACAM3 results to phosporylation of its ITAM by protein kinases of the Src family; in turn, a signal transduction pathway is initiated that leads to bacterial engulfment and killing (Hauck et al. 1998; McCaw et al. 2003). On the other hand, it was shown that the presence of the ITIM of CEACAM1 was essential to suppress adaptive immune response upon bacterial infection of the genus *Neisseria* (Boulton and Gray-Owen 2002). Moreover, CEACAM1, as opposed to CEACAM3, acts as a tumor suppressor, shown to inhibit the growth of prostate, colon, and breast tumors (Estrera et al. 2001; Volpert et al. 2002; Sappino et al. 2012). The ITIM could presumably account for CEACAM1’s tumor suppressive properties. In this study, an ITAM was also detected in the cytoplasmic tail of CEACAM19, which is overexpressed in several types of cancer (Scorilas et al. 2003; Michaelidou et al. 2013). The oncogenic potential of CEACAM19 may, at least partially, depend on the presence of the ITAM. Further experimental studies could probably verify the signaling regulatory role of ITAM/ITIM in various cellular activities.

We expect that the findings of our study could lay the foundation for the design of experimental studies directed toward the elucidation of the biochemical function of the putative *CEA* and *CEA*-encoding proteins, taking into consideration the identified protein patterns. The conserved amino acids, also, detected in the protein sequences could represent potential drug targets and should be considered in light of their exploitation in the design of therapeutic agents in anticancer research.

## Supplementary Material

Supplementary table S1 and figures S1 and S2 are available at *Genome Biology and Evolution* online (http://www.gbe.oxfordjournals.org/).

Supplementary Data
