# Supplementary material for: A Comprehensive Phylogenetic and Structural Analysis of the Carcinoembryonic Antigen (CEA) Gene Family
Source: Genome Biol Evol. 2014 May 23;6(6):1314–26. doi: 10.1093/gbe/evu103 (PMC4079198; doi:10.1093/gbe/evu103)
Supplement: Supplementary Data [file supp_6_6_1314__index.html]

“A comprehensive phylogenetic and structural analysis of the carcinoembryonic antigen (CEA) gene family” — A Comprehensive Phylogenetic and Structural Analysis of the Carcinoembryonic Antigen (CEA) Gene Family — Supplementary Data 

# A Comprehensive Phylogenetic and Structural Analysis of the Carcinoembryonic Antigen (CEA) Gene Family

## Supplementary Data

files

**Files in this Data Supplement:**

- Supplementary Data - zip file
