# Supplementary material for: A Comprehensive Phylogenetic and Structural Analysis of the Carcinoembryonic Antigen (CEA) Gene Family
Source: Genome Biol Evol. 2014 May 23;6(6):1314–26. doi: 10.1093/gbe/evu103 (PMC4079198; doi:10.1093/gbe/evu103)
Supplement: Supplementary Data [file supp_evu103_suppl_data.zip › SupplementaryTable_1.docx]

| Organism | Sequence | Accession code |
| --- | --- | --- |
| *Homo sapiens* | HumanCEACAM21 | Q3KPI0^1^, ENSP00000385739^2^, NP_001091976.1^3^ |
| *Homo sapiens* | HumanCEACAM4 | O75871^1^, ENSP00000221954^2^, NP_001808.2^3^ |
| *Homo sapiens* | HumanCEACAM7 | Q14002^1^, ENSP00000385932^2^, NP_008821.1^3^ |
| *Homo sapiens* | HumanCEACAM5 | P06731^1^, ENSP00000221992^2^, NP_004354.2^3^ |
| *Homo sapiens* | HumanCEACAM6 | P40199^1^, ENSP00000199764^2^, NP_002474.3^3^ |
| *Homo sapiens* | HumanCEACAM3 | P40198^1^, ENSP00000349971^2^, NP_001806.2^3^ |
| *Homo sapiens* | HumanCEACAM1 | P13688^1^, ENSP00000161559^2^, NP_001703.2^3^ |
| *Homo sapiens* | HumanCEACAM8 | P31997^1^, ENSP00000244336^2^, NP_001807.2^3^ |
| *Homo sapiens* | HumanPSG3 | Q16557^1^, ENSP00000332215^2^, NP_066296.2^3^ |
| *Homo sapiens* | HumanPSG8 | Q9UQ74^1^, ENSP00000305005^2^, NP_874366.1^3^ |
| *Homo sapiens* | HumanPSG1 | ENSP00000244296^2^, NP_008836.2^3^ |
| *Homo sapiens* | HumanPSG6 | Q00889^1^, ENSP00000292125^2^, NP_002773.1^3^ |
| *Homo sapiens* | HumanPSG11 | Q9UQ72^1^, ENSP00000319140^2^, NP_002776.3^3^ |
| *Homo sapiens* | HumanPSG2 | P11465^1^, ENSP00000385706^2^, NP_112536.2^3^ |
| *Homo sapiens* | HumanPSG5 | Q15238^1^, ENSP00000382334^2^, NP_001123486.1^3^ |
| *Homo sapiens* | HumanPSG4 | Q00888^1^, ENSP00000384770^2^, NP_002771.2^3^ |
| *Homo sapiens* | HumanPSG9 | Q00887^1^, ENSP00000270077^2^, NP_002775.3^3^ |
| *Homo sapiens* | HumanCEACAM20 | A7LFK7^1^, NP_001096067.1^3^ |
| *Homo sapiens* | HumanIGSF23 | A1L1A6^1^, ENSP00000385592^2^,A1L1A6^3^ |
| *Homo sapiens* | HumanPVR | P15151^1^, ENSP00000402060^2^, P15151^3^ |
| *Homo sapiens* | HumanCEACAM19 | Q7Z692^1^, ENSP00000384887^2^, NP_064604.2^3^ |
| *Homo sapiens* | HumanCEACAM16 | Q2WEN9^1^, ENSP00000385576^2^, NP_001034302.2^3^ |
| *Homo sapiens* | HumanSIGLEC8 | Q9NYZ4^1^, ENSP00000321077^2^, Q9NYZ4^3^ |
| *Homo sapiens* | HumanCEACAM18 | A8MTB9^1^, ENSP00000402203^2^, NP_001073874.1^3^ |
| *Homo sapiens* | HumanSIGLEC12 | Q96PQ1^1^, ENSP00000291707^2^, Q96PQ1^3^ |
| *Pan troglodytes* | ChimpanzeeCEACAM4 | H2QGE4^1^, ENSPTRP00000018939^2^ |
| *Pan troglodytes* | ChimpanzeeCEACAM7 | H2QGE5^1^, ENSPTRP00000018942^2^, XP_003316413.1^3^ |
| *Pan troglodytes* | ChimpanzeeCEACAM5 | H2QGE6^1^, ENSPTRP00000018943^2^, XP_003316414.1^3^ |
| *Pan troglodytes* | ChimpanzeeCEACAM6 | H2QGH0^1^, ENSPTRP00000018996^2^, XP_003316416.1^3^ |
| *Pan troglodytes* | ChimpanzeeCEACAM3 | H2QGE7^1^, ENSPTRP00000018946^2^, XP_003316417.1^3^ |
| *Pan troglodytes* | ChimpanzeeCEACAM1 | H2QGG8^1^, ENSPTRP00000018992^2^ |
| *Pan troglodytes* | ChimpanzeeCEACAM8 | H2R427^1^, ENSPTRP00000046471^2^, XP_512705.2^3^ |
| *Pan troglodytes* | ChimpanzeePSG3 | H2QGH1^1^ , ENSPTRP00000019018^2^ |
| *Pan troglodytes* | ChimpanzeePSG8 | H2RFY8^1^, ENSPTRP00000060434^2^, XP_003316439.2^3^ |
| *Pan troglodytes* | ChimpanzeePSG1 | H2RG18^1^, ENSPTRP00000060464^2^, XP_512709.3^3^ |
| *Pan troglodytes* | ChimpanzeePSG11 | H2R0Z2^1^, ENSPTRP00000041481^2^ |
| *Pan troglodytes* | ChimpanzeePSG2 | H2RCX9^1^, ENSPTRP00000057986^2^ |
| *Pan troglodytes* | ChimpanzeePSG9 | H2QGH2^1^, ENSPTRP00000019019^2^ |
| *Pan troglodytes* | ChimpanzeePSG5 | H2R621^1^, ENSPTRP00000048662^2^ |
| *Pan troglodytes* | ChimpanzeePSG4 | H2RBD8^1^, ENSPTRP00000055531^2^, XP_003339369.1^3^ |
| *Pan troglodytes* | ChimpanzeeIGSF23 | A1L1A6^1^, ENSPTRP00000055638^2^, XP_512735^3^ |
| *Pan troglodytes* | ChimpanzeePVR | H2RBG0^1^, ENSPTRP00000019082^2^, JAA23386^3^ |
| *Pan troglodytes* | ChimpanzeeCEACAM19 | H2QGK0^1^, ENSPTRP00000019084^2^, XP_003316519.1^3^ |
| *Pan troglodytes* | ChimpanzeeCEACAM16 | H2RCB2^1^, ENSPTRP00000057043^2^ |
| *Pan troglodytes* | ChimpanzeeSIGLEC8 | H2QH05^1^, ENSPTRP00000019556^2^ |
| *Pan troglodytes* | ChimpanzeeCEACAM18 | H2RD66^1^, ENSPTRP00000058394^2^ |
| *Pan troglodytes* | ChimpanzeeSIGLEC12 | Q95LH0^1^, ENSPTRP00000048511^2^, NP_001009040^3^ |
| *Pongo abelii* | OrangutanCEACAM21 | H2NYY2^1^, ENSPPYP00000011215^2^, XP_002829319.1^3^ |
| *Pongo abelii* | OrangutanCEACAM4 | H2NYY3^1^, ENSPPYP00000011216^2^ |
| *Pongo abelii* | OrangutanCEACAM7 | H2NYY4^1^, ENSPPYP00000011217^2^ |
| *Pongo abelii* | OrangutanCEACAM5 | H2NYY5^1^, ENSPPYP00000011218^2^ |
| *Pongo abelii* | OrangutanCEACAM6 | H2NYY6^1^, ENSPPYP00000011219^2^ |
| *Pongo abelii* | OrangutanCEACAM3 | H2NYY8^1^, ENSPPYP00000011221^2^ |
| *Pongo abelii* | OrangutanCEACAM1 | H2NZ13^1^, ENSPPYP00000011247^2^ |
| *Pongo abelii* | OrangutanCEACAM8 | H2NZ15^1^, ENSPPYP00000011249^2^ |
| *Pongo abelii* | OrangutanPSG9 | H2NZ16^1^,ENSPPYP00000011250^2^ |
| *Pongo abelii* | OrangutanPSG5 | H2NZ17^1^, ENSPPYP00000011251^2^ |
| *Pongo abelii* | OrangutanPSG3 | H2P0K1^1^, ENSPPYP00000011801^2^ |
| *Pongo abelii* | OrangutanPSG1 | H2P0L8^1^, ENSPPYP00000011819^2^ |
| *Pongo abelii* | OrangutanCEACAM20 | H2NZ56^1^, ENSPPYP00000011290^2^ |
| *Pongo abelii* | OrangutanPVR | H2NZ57^1^, ENSPPYP00000011291^2^, XP_002829419^3^ |
| *Pongo abelii* | OrangutanCEACAM19 | H2NZ58^1^, ENSPPYP00000011292^2^ |
| *Pongo abelii* | OrangutanCEACAM16 | H2NZ59^1^, ENSPPYP00000011293^2^ |
| *Pongo abelii* | OrangutanSIGLEC8 | H2NZW4^2^, ENSPPYP00000011558^2^, XP_003780631^3^ |
| *Pongo abelii* | OrangutanCEACAM18 | H2NZW5^1^, ENSPPYP00000011559^2^ |
| *Pongo abelii* | OrangutanSIGLEC12 | H2P0P9^1^, ENSPPYP00000011852^2^, XP_002834559^3^ |
| *Macaca mulatta* | MacaqueCEACAM4 | F7ECD5^1^, ENSMMUP00000023790^2^, XP_001102867.1^3^ |
| *Macaca mulatta* | MacaqueCEACAM7 | F7ECC0^1^, ENSMMUP00000023793^2^ |
| *Macaca mulatta* | MacaqueCEACAM5 | F6YVT9^1^, ENSMMUP00000017645^2^ |
| *Macaca mulatta* | MacaqueCEACAM6 | F7BJG1^1^, ENSMMUP00000025906^2^ |
| *Macaca mulatta* | MacaqueCEACAM3 | F6Z2M1^1^, ENSMMUP00000017628^2^ |
| *Macaca mulatta* | MacaqueCEACAM1 | F6YVS0^1^, ENSMMUP00000017647^2^ |
| *Macaca mulatta* | MacaqueCEACAM8 | F6X7W3^1^, ENSMMUP00000017365^2^ |
| *Macaca mulatta* | MacaquePSG | H9H3H6^1^, ENSMMUP00000015035^2^ |
| *Macaca mulatta* | MacaqueIGSF23 | F6Z2F5^1^, ENSMMUP00000031574^2^ |
| *Macaca mulatta* | MacaquePVR | Q0MSE6^1^, ENSMMUP00000004136^2^, NP_001036851^3^ |
| *Macaca mulatta* | MacaqueCEACAM19 | F6QMB9^1^, ENSMMUP00000013415^2^ |
| *Macaca mulatta* | MacaqueCEACAM16 | F6TG62^1^, ENSMMUP00000013124^2^ |
| *Macaca mulatta* | MacaqueSIGLEC10 | F7GW74^1^, ENSMMUP00000027786^2^, XP_001116352^3^ |
| *Macaca mulatta* | MacaqueCEACAM18 | F7A4I9^1^, ENSMMUP00000029580^2^ |
| *Macaca mulatta* | MacaqueSIGLEC6 | F7EIR9^1^, ENSMMUP00000016770^2^ |
| *Callithrix jacchus* | MarmosetCEACAM7 | F6VFI5^1^, ENSCJAP00000037576^2^ |
| *Callithrix jacchus* | MarmosetCEACAM5 | F7HXU0^1^, ENSCJAP00000052302^2^ |
| *Callithrix jacchus* | MarmosetCEACAM8 | F7D4I0^1^, ENSCJAP00000023829^2^ |
| *Callithrix jacchus* | MarmosetPSG | F7IRU1^1^, ENSCJAP00000031376^2^ |
| *Callithrix jacchus* | MarmosetPVR | F7GTL9^1^, ENSCJAP00000024048^2^ |
| *Callithrix jacchus* | MarmosetCEACAM19 | F7GEH9^1^,ENSCJAP00000024235^2^ |
| *Callithrix jacchus* | MarmosetCEACAM16 | F7GN3^1^2,ENSCJAP00000024213^2^ |
| *Callithrix jacchus* | MarmosetSIGLEC10 | F6X015^1^, ENSCJAP00000034369^2^ |
| *Callithrix jacchus* | MarmosetCEACAM18 | F6Z9E3^1^, ENSCJAP00000034320^2^ |
| *Mus musculus* | MouseCeacam15 | ENSMUSP00000104138^2^, NP_780524.1^3^ |
| *Mus musculus* | MouseCeacam9 | Q78T27^1^, ENSMUSP00000001984^2^, NP_036057.1^3^ |
| *Mus musculus* | MousePsg16 | D0VY58^1^, ENSMUSP00000113025^2^, NP_031702.3^3^ |
| *Mus musculus* | MouseCeacam3 | L7N1Y1^1^, ENSMUSP00000069892^2^, NP_473400.1^3^ |
| *Mus musculus* | MousePsg29 | Q3URN6^1^,ENSMUSP00000075320^2^, NP_473405.1^3^ |
| *Mus musculus* | MouseCeacam5 | Q3UKK2^1^, ENSMUSP00000080582^2^, NP_082756.1^3^ |
| *Mus musculus* | MouseCeacam14 | Q78Y72^1^, ENSMUSP00000023953^2^, NP_080233.1^3^ |
| *Mus musculus* | MouseGm5155 | Q3UKC7^1^, ENSMUSP00000104130^2^ |
| *Mus musculus* | MouseCeacam11 | Q9D0Z8^1^, ENSMUSP00000092393^2^, NP_075778.2^3^ |
| *Mus musculus* | MouseCeacam13 | Q9DAY4^1^, ENSMUSP00000104128^2^, NP_081486.1^3^ |
| *Mus musculus* | MouseCeacam12 | Q3UKP4^1^, ENSMUSP00000032520^2^, NP_080363.2^3^ |
| *Mus musculus* | MousePsg18 | B2RSG7^1^, ENSMUSP00000003597^2^, NP_036093.2^3^ |
| *Mus musculus* | MousePsg28 | Q4KL66^1^, ENSMUSP00000019291^2^, NP_473404.3^3^ |
| *Mus musculus* | MousePsg26 | Q4KL65^1^, ENSMUSP00000092392^2^, NP_001025064.1^3^ |
| *Mus musculus* | MousePsg25 | Q497W1^1^, ENSMUSP00000092389^2^, NP_473401.1^3^ |
| *Mus musculus* | MousePsg27 | Q497W2^1^, ENSMUSP00000092388^2^, NP_001032245.1^3^ |
| *Mus musculus* | MousePsg23 | Q9D2U0^1^, ENSMUSP00000056586^2^, NP_064657.2^3^ |
| *Mus musculus* | MousePsg21 | Q9DAV5^1^, ENSMUSP00000092387^2^, NP_081679.2^3^ |
| *Mus musculus* | MousePsg20 | E9Q9B4^1^, ENSMUSP00000075973^2^, NP_473399.1^3^ |
| *Mus musculus* | MousePsg22 | Q810J1^1^, ENSMUSP00000050633^2^, NP_001004152.1^3^ |
| *Mus musculus* | MousePsg19 | Q4KL31^1^, ENSMUSP00000004657^2^, NP_036094.2^3^ |
| *Mus musculus* | MousePsg17 | Q62056^1^, ENSMUSP00000004655^2^, NP_031703.1^3^ |
| *Mus musculus* | MouseCeacam16 | E9QA28^1^, ENSMUSP00000014830^2^, NP_001028591.2^3^ |
| *Mus musculus* | MouseCeacam19 | Q3TQ88^1^, ENSMUSP00000057433^2^, NP_796010.1^3^ |
| *Mus musculus* | MousePvr | Q8K094^1^, ENSMUSP00000039205^2^, NP_081790^3^ |
| *Mus musculus* | MouseIgsf23 | B2RTN2^1^, ENSMUSP00000047914^2^, NP_081584^3^ |
| *Mus musculus* | MousePr17a-ps8 | ENSMUSP00000092345^2^ |
| *Mus musculus* | MouseCeacam20 | Q9D2Z1^1^, ENSMUSP00000092344^2^, NP_082115.2^3^ |
| *Mus musculus* | MouseCeacam1 | P31809^1^, ENSMUSP00000096266^2^, NP_001034274.1^3^ |
| *Mus musculus* | MouseCeacam2 | Q925P2^1^, ENSMUSP00000048118^2^, NP_031569.1^3^ |
| *Mus musculus* | MouseCeacam18 | Q9D871^1^, ENSMUSP00000032663^2^, NP_082512.1^3^ |
| *Mus musculus* | MouseSiglece | Q91Y57^1^, ENSMUSP00000032667^2^, NP_112458^3^ |
| *Rattus norvegicus* | RatCeacam15 | D4ADA7^1^, ENSRNOP00000040118^2^ |
| *Rattus norvegicus* | RatCea1 | F1M035^1^, ENSRNOP00000054957^2^ |
| *Rattus norvegicus* | RatCeacam9 | Q9R121^1^, ENSRNOP00000066209^2^ |
| *Rattus norvegicus* | RatCgm4 | Q4V8J0^1^, ENSRNOP00000033854^2^, NP_036657.1^3^ |
| *Rattus norvegicus* | RatPsg16 | Q4V883^1^, ENSRNOP00000054868^2^, NP_001020850.1^3^ |
| *Rattus norvegicus* | RatCeacam3 | Q4KLZ7^1^, ENSRNOP00000023663^2^, NP_036834.2^3^ |
| *Rattus norvegicus* | RatPsgb1 | G3V925^1^, ENSRNOP00000034066^2^ |
| *Rattus norvegicus* | RatPsg29 | Q4V8K0^1^, ENSRNOP00000044708^2^, NP_001020812.1^3^ |
| *Rattus norvegicus* | RatCea2 | D3ZAN0^1^, ENSRNOP00000024155^2^ |
| *Rattus norvegicus* | RatCea3 | D3ZT23^1^, ENSRNOP00000054849^2^ |
| *Rattus norvegicus* | RatCeacam11 | Q4QQU3^1^, ENSRNOP00000023686^2^, NP_001020575.1^3^ |
| *Rattus norvegicus* | RatPsg19 | Q4V8L4^1^, ENSRNOP00000054798^2^ |
| *Rattus norvegicus* | RatCeacam16 | D3ZQE1^1^, ENSRNOP00000041331^2^ |
| *Rattus norvegicus* | RatCeacam19 | D3ZE93^1^, ENSRNOP00000043820^2^, NP_001185899.1^3^ |
| *Rattus norvegicus* | RatPvr | E9PTC0^1^, ENSRNOP00000044638^2^ |
| *Rattus norvegicus* | RatIgsf23 | M0R9H9^1^, ENSRNOP00000066148^2^ |
| *Rattus norvegicus* | RatCeacam20 | D4ADQ8^1^, ENSRNOP00000026060^2^ |
| *Rattus norvegicus* | RatCeacam10 | Q64724^1^, ENSRNOP00000027213^2^, NP_775461.1^3^ |
| *Rattus norvegicus* | RatCeacam6 | F7FIX1^1^, ENSRNOP00000054410^2^ |
| *Rattus norvegicus* | RatCeacam1 | P16573^1^, ENSRNOP00000046654^2^ |
| *Rattus norvegicus* | RatCeacam18 | D3ZXU3^1^, ENSRNOP00000032251^2^ |
| *Rattus norvegicus* | RatSiglece | F1M8J5^1^, ENSRNOP00000040647^2^ |
| *Bos taurus* | CowCeacam1 | Q6VAN8^1^, ENSBTAP00000009007^2^, NP_991357.1^3^ |
| *Bos taurus* | CowCeacam5 | E1BMH6^1^, ENSBTAP00000007381^2^ |
| *Bos taurus* | CowCeacam20 | F1N2U9^1^, ENSBTAP00000014658^2^ |
| *Bos taurus* | CowIgsf23 | G3MY26^1^, ENSBTAP00000054452^2^ |
| *Bos taurus* | CowCeacam19 | F6RLN7^1^, ENSBTAP00000020358^2^ |
| *Bos taurus* | CowCeacam16 | E1BKM1^1^, ENSBTAP00000019915^2^ |
| *Bos taurus* | CowCeacam18 | F1MU33^1^, ENSBTAP00000006050^2^ |
| *Equus caballus* | HorseCea1 | F6PZP5^1^, ENSECAP00000008127^2^ |
| *Equus caballus* | HorseCea2 | F6T1X0^1^, ENSECAP00000022952^2^ |
| *Equus caballus* | HorseCea3 | F7DZU8^1^, ENSECAP00000017975^2^ |
| *Equus caballus* | HorseCea4 | F6Z600^1^, ENSECAP00000007152^2^ |
| *Equus caballus* | HorseCea5 | F7DNF6^1^, ENSECAP00000012555^2^ |
| *Equus caballus* | HorseCeacam21 | F7BL07^1^, ENSECAP00000016872^2^ |
| *Equus caballus* | HorseCea6 | F6VCS1^1^, ENSECAP00000001275^2^ |
| *Equus caballus* | HorsePvr | F6YCJ3^1^, ENSECAP00000007218^2^ |
| *Equus caballus* | HorseCeacam19 | F7C1Z7^1^, ENSECAP00000008937^2^ |
| *Equus caballus* | HorseCeacam16 | F6RDS1^1^, ENSECAP00000010360^2^ |
| *Canis familiaris* | DogCeacam18 | E2QYA9^1^, ENSCAFP00000004242^2^ |
| *Canis familiaris* | DogCeacam16 | E2RCH4^1^, ENSCAFP00000006937^2^ |
| *Canis familiaris* | DogCeacam19 | XP_003432705.1^3^ |
| *Canis familiaris* | DogPvr | F1PVS7^1^, ENSCAFP00000006945^2^, XP_005616524^3^ |
| *Canis familiaris* | DogIgsf23 | E2RCE8^1^, ENSCAFP00000006946^2^ |
| *Canis familiaris* | DogCeacam20 | E2RPZ7^1^, ENSCAFP00000032422^2^ |
| *Canis familiaris* | DogCeacam23 | A1YV60^1^, NP_001091021.1^3^ |
| *Canis familiaris* | DogCeacam24 | A1YV61^1^, ENSCAFP00000042712^2^, NP_001091023.1^3^ |
| *Canis familiaris* | DogCeacam25 | A1YV62^1^, ENSCAFP00000041423^2^, NP_001106930.1^3^ |
| *Canis familiaris* | DogCeacam28 | A1YV64^1^, ENSCAFP00000007154^2^, NP_001091015.1^3^ |
| *Canis familiaris* | DogCeacam1 | Q004B2^1^, ENSCAFP00000007181^2^, NP_001091026.1^3^ |
| *Canis familiaris* | DogCeacam5 | E2R9W0^1^, ENSCAFP00000007180^2^ |
| *Dasypus novemcinctus* | ArmadilloCea1 | ENSDNOP00000015686^2^ |
| *Dasypus novemcinctus* | ArmadilloCea2 | ENSDNOP00000010007^2^ |
| *Dasypus novemcinctus* | ArmadilloCea3 | ENSDNOP00000002630^2^ |
| *Dasypus novemcinctus* | ArmadilloCea4 | ENSDNOP00000016276^2^ |
| *Dasypus novemcinctus* | ArmadilloCeacam20 | ENSDNOP00000017322^2^ |
| *Dasypus novemcinctus* | ArmadilloCeacam16 | ENSDNOP00000016463^2^ |
| *Dasypus novemcinctus* | ArmadilloCeacam18 | ENSDNOP00000017623^2^ |
| *Loxodonta africana* | ElephantCea1 | G3TTV3^1^, ENSLAFP00000019011^2^ |
| *Loxodonta africana* | ElephantCea2 | G3U8C4^1^, ENSLAFP00000024082^2^ |
| *Loxodonta africana* | ElephantCea3 | G3TIY3^1^, ENSLAFP00000014648^2^ |
| *Loxodonta africana* | ElephantPvr | G3T614^1^, ENSLAFP00000008923^2^ |
| *Loxodonta africana* | ElephantCeacam19 | G3T772^1^, ENSLAFP00000009428^2^ |
| *Loxodonta africana* | ElephantCeacam16 | G3TLR7^1^, ENSLAFP00000015904^2^ |
| *Monodelphis domestica* | OpossumCea1 | F7DAN1^1^, ENSMODP00000011571^2^ |
| *Monodelphis domestica* | OpossumCea2 | F7F9J6^1^, ENSMODP00000011877^2^ |
| *Monodelphis domestica* | OpossumCea3 | F7EUJ0^1^, ENSMODP00000023879^2^ |
| *Monodelphis domestica* | OpossumCea4 | K7E183^1^, ENSMODP00000039534^2^ |
| *Monodelphis domestica* | OpossumCea5 | F6XT90^1^, ENSMODP00000012142^2^ |
| *Monodelphis domestica* | OpossumCea6 | F6S0V2^1^, ENSMODP00000012003^2^ |
| *Monodelphis domestica* | OpossumCea7 | K7E317^1^, ENSMODP00000040168^2^ |
| *Monodelphis domestica* | OpossumCea8 | F6R843^1^, ENSMODP00000012441^2^ |
| *Monodelphis domestica* | OpossumCea9 | K7E4G5^1^, ENSMODP00000040667^2^ |
| *Monodelphis domestica* | OpossumCea10 | F6QMW6^1^, ENSMODP00000012454^2^ |
| *Monodelphis domestica* | OpossumCea11 | F6Y7Z8^1^, ENSMODP00000012481^2^ |
| *Monodelphis domestica* | OpossumCea12 | F6U9R9^1^, ENSMODP00000012559^2^ |
| *Monodelphis domestica* | OpossumCeacam20 | F6QG81^1^, ENSMODP00000026094^2^ |
| *Monodelphis domestica* | OpossumPvr | F6QG96^1^, ENSMODP00000026092^2^ |
| *Monodelphis domestica* | OpossumCeacam19 | F6QGA4^1^, ENSMODP00000026091^2^ |
| *Monodelphis domestica* | OpossumCeacam16 | F6QGB2^1^, ENSMODP00000026090^2^ |
| *Ornithorhynchus anatinus* | Platypus Cea1 | F7G7V0^1^, ENSOANP00000026251^2^ |
| *Ornithorhynchus anatinus* | Platypus Cea2 | F6RZB7^1^, ENSOANP00000015399^2^ |
| *Ornithorhynchus anatinus* | Platypus Cea3 | F7AMC7^1^, ENSOANP00000015401^2^ |
| *Ornithorhynchus anatinus* | Platypus Cea4 | F7AM87^1^, ENSOANP00000015405^2^ |
| *Ornithorhynchus anatinus* | PlatypusPvrl2 | F7DCN5^1^, ENSOANP00000019287^2^ |
| *Ornithorhynchus anatinus* | Platypus Ceacam16 | F7DCL4^1^, ENSOANP00000019290^2^ |
| *Anolis carolinensis* | LizardCeacam20 | H9GCC8^1^, ENSACAP00000007004^2^ |
| *Anolis carolinensis* | LizardCeacam19 | scaffold:AnoCar2.0:GL343707.1:12466:13503:-1^2a^ |
| *Xenopus tropicalis* | FrogCea1 | F7B1E8^1^, ENSXETP00000060297^2^ |
| *Xenopus tropicalis* | FrogCea2 | L7N404^1^, ENSXETP00000055007^2^ |
| *Xenopus tropicalis* | FrogCea3 | L7N406^1^, ENSXETP00000055010^2^ |
| *Xenopus tropicalis* | FrogCea4 | F6XW14^1^, ENSXETP00000033868^2^ |
| *Xenopus tropicalis* | FrogCea5 | K9J846^1^, ENSXETP00000026295^2^ |
| *Xenopus tropicalis* | FrogCea6 | L7N405^1^, ENSXETP00000055009^2^ |
| *Xenopus tropicalis* | FrogCea7 | L7N3I7^1^, ENSXETP00000033857^2^ |
| *Xenopus tropicalis* | FrogCea8 | F7AEF9^1^, ENSXETP00000061615^2^ |
| *Xenopus tropicalis* | FrogCea9 | F6UF30^1^, ENSXETP00000041880^2^ |
| *Xenopus tropicalis* | FrogCea10 | F6UF08^1^, ENSXETP00000041889^2^ |
| *Xenopus tropicalis* | FrogCea11 | F6UEX7^1^, ENSXETP00000041897^2^ |
| *Xenopus tropicalis* | FrogCea12 | F6XVD8^1^, ENSXETP00000033871^2^ |
| *Xenopus tropicalis* | FrogCea13 | F6TUQ0^1^, ENSXETP00000044985^2^ |
| *Danio rerio* | ZebrafishGrik5(1) | E7FB75^1^, ENSDARP00000106550^2^ |
| *Danio rerio* | ZebrafishCea1 | A4JYL4^1^, ENSDARP00000101845^2^, NP_001091716.1^3^ |
| *Danio rerio* | ZebrafishCea2 | F1R3B4^1^, ENSDARP00000116248^2^ |
| *Danio rerio* | ZebrafishCea3 | F1QX85^1^, ENSDARP00000113934^2^ |
| *Danio rerio* | ZebrafishCea4 | F1QT52^1^, ENSDARP00000122704^2^ |
| *Danio rerio* | ZebrafishCea5 | F1QGB2^1^, ENSDARP00000123071^2^ |
| *Danio rerio* | ZebrafishCea6 | F1QSL7^1^, ENSDARP00000122087^2^ |
| *Danio rerio* | ZebrafishCea7 | E9QBN5^1^, ENSDARP00000116462^2^ |
| *Danio rerio* | ZebrafishCea8 | F1R4F9^1^, ENSDARP00000060265^2^, NP_001107266.1^3^ |
| *Danio rerio* | ZebrafishCea9 | E9QJI4^1^, ENSDARP00000115706^2^ |
| *Danio rerio* | ZebrafishGrik5(2) | F1R276^1^, ENSDARP00000104383^2^ |
| *Takifugu rubripes* | PufferfishCea1 | H2SFB4^1^, ENSTRUP00000011095^2^ |
| *Takifugu rubripes* | PufferfishCea2 | H2SGL3^1^, ENSTRUP00000011545^2^ |
| *Takifugu rubripes* | PufferfishCea3 | H2SHA2^1^, ENSTRUP00000011784^2^ |
| *Takifugu rubripes* | PufferfishGrik5(1) | H2SJS1^1^, ENSTRUP00000012654^2^ |
| *Takifugu rubripes* | PufferfishGrik5(2) | H2S5B1^1^, ENSTRUP00000007583^2^ |
| *Takifugu rubripes* | PufferfishCea4 | H2S7R6^1^, ENSTRUP00000008438^2^ |
| *Takifugu rubripes* | PufferfishGrik5(2) | H2MDW8^1^, ENSORLP00000016770^2^ |
| *Oryzias latipes* | MedakaCea | H2MDY7^1^, ENSORLP00000016789^2^ |
| *Leucoraja erinacea* | LIttleSkate | FF601895^3b^ |

^1^UniProtKB (<http://www.uniprot.org/>), ^2^ENSEMBL (<http://www.ensembl.org/>); ^3^NCBI’s RefSeq (<http://www.ncbi.nlm.nih.gov/refseq/>); ^a^translated nucleotide sequence; ^b^translated expressed sequence tag sequence
